# GPX4 and FSP1 Expression in Lung Adenocarcinoma: Prognostic Implications and Ferroptosis-Based Therapeutic Strategies

**DOI:** 10.3390/cancers16223888

**Published:** 2024-11-20

**Authors:** Hirotomo Takahara, Takumi Kanazawa, Haruna Oshita, Yoshinobu Tomita, Yuri Hananoi, Sachiko Ishibashi, Masumi Ikeda, Asuka Furukawa, Mayumi Kinoshita, Kurara Yamamoto, Yuki Kato, Hironori Ishibashi, Kenichi Okubo, Morito Kurata, Masanobu Kitagawa, Kenichi Ohashi, Kouhei Yamamoto

**Affiliations:** 1 Department of Thoracic Surgery, Graduate School of Medical and Dental Sciences, Tokyo Medical and Dental University, Tokyo 113-8510, Japan; tkhrhrtm.0622.thsr@tmd.ac.jp (H.T.); hishiba.thsr@tmd.ac.jp (H.I.); okubo.thsr@tmd.ac.jp (K.O.); 2 Department of Clinical Laboratory Medicine, Faculty of Health Science Technology, Bunkyo Gakuin University, Tokyo 113-8668, Japan; 23ms203@s.bgu.ac.jp (T.K.); mkinoshita@bgu.ac.jp (M.K.); 3 Department of Human Pathology, Graduate School of Medical and Dental Sciences, Tokyo Medical and Dental University, Tokyo 113-8510, Japan; 210020ms@tmd.ac.jp (H.O.); 18cm127@s.bgu.jp (Y.T.); 210068ms@tmd.ac.jp (Y.H.); a.tajima.pth1@tmd.ac.jp (A.F.); kakipth1@tmd.ac.jp (K.Y.); ykatpth1@tmd.ac.jp (Y.K.); kohashi.pth1@tmd.ac.jp (K.O.); 4 Department of Comprehensive Pathology, Graduate School of Medical and Dental Sciences, Tokyo Medical and Dental University, Tokyo 113-8510, Japan; sishpth2@tmd.ac.jp (S.I.); mikepth2@tmd.ac.jp (M.I.); kurata.pth2@tmd.ac.jp (M.K.); masa.pth2@tmd.ac.jp (M.K.)

**Keywords:** lung adenocarcinoma, ferroptosis, 4-HNE, GPX4, FSP1

## Abstract

**Background:** Primary lung cancer is among the cancers with the poorest prognosis, having the highest mortality rate among men and the second highest among women in Japan. While surgery is the primary treatment, advanced stages often require pharmacotherapy. Recently, ferroptosis, an iron-dependent form of cell death caused by lipid peroxidation, has gained attention as a potential therapeutic strategy. This study investigated the prognostic impact of lipid peroxidation marker and regulators involved in ferroptosis in lung adenocarcinoma. **Methods**: We analyzed 207 patients who underwent resection surgery for lung adenocarcinoma at Tokyo Medical and Dental University Hospital. Immunohistochemistry was used to evaluate the expression levels of glutathione peroxidase 4 (GPX4), ferroptosis suppressor protein 1 (FSP1), and 4-hydroxy-2-nonenal (4-HNE). The association between these markers and clinicopathological factors was assessed, and in vitro experiments were conducted to examine the effects of these markers on cell death. **Results**: Low cytoplasmic accumulation of 4-HNE and low expression of GPX4 were associated with a worse prognosis, and low FSP1 expression was associated with unfavorable relapse-free survival. In vitro experiments demonstrated that 4-HNE inhibited cell proliferation, and combined inhibition of GPX4 and FSP1 induced ferroptosis. **Conclusions**: These findings suggest that lipid peroxidation markers and regulators can serve as prognostic biomarkers and therapeutic targets in lung adenocarcinoma.

## Simple Summary

This study was proposed to address the poor prognosis of primary lung cancer, particularly lung adenocarcinoma, which remains a leading cause of cancer-related mortality. The authors aim to explore the prognostic impact of lipid peroxidation markers and regulators involved in ferroptosis, an emerging form of cell death, to identify potential therapeutic targets. By analyzing the expression levels of glutathione peroxidase 4 (GPX4), ferroptosis suppressor protein 1 (FSP1), and 4-Hydroxy-2-nonenal (4-HNE) in resected lung adenocarcinoma tissues, the study seeks to establish a relationship with clinicopathological factors and patient outcomes. The findings revealed that low 4-HNE and low GPX4 expression are associated with worse prognosis, while low FSP1 expression correlates with unfavorable relapse-free survival. These insights suggest that lipid peroxidation markers and ferroptosis regulators could serve as valuable prognostic biomarkers and therapeutic targets. This research has the potential to significantly impact the scientific community by guiding the development of novel, targeted therapies for lung adenocarcinoma, ultimately improving patient outcomes.

## 1. Introduction

Primary lung cancer is one of the cancers with the poorest prognosis. According to a 2019 report, it has the highest mortality rate by site among cancers in Japan, ranking first among men and second among women [1]. It is also the third and fourth most common cancer among men and women in Japan, respectively. Thus, further exploration of treatment methods to improve the prognosis of patients with lung cancer is crucial. Surgery is the first choice of treatment, but pharmacotherapy is used for patients with advanced stages of cancer or those who cannot tolerate surgery due to comorbidities. Among drug therapies, anticancer drugs, such as platinum-based drugs, are the most commonly used to induce apoptosis in cancer cells and kill them. On the other hand, in recent years, drugs such as molecularly targeted drugs and immune checkpoint inhibitors, which treat cancer through mechanisms different from those of traditional anticancer drugs, have been researched and developed and are now being utilized in clinical practice.

One of the most studied cancer therapies in recent years is ferroptosis, a process characterized by iron-dependent accumulation of lipid peroxides that induces cell death. 4-Hydroxy-2-nonenal (4-HNE) is one of the metabolites produced by the metabolism and degradation of lipid peroxide and has a variety of physiological activities [2]. 4-HNE also plays an important role in the development and progression of malignant tumors through mechanisms such as altering intracellular signaling [3]. Furthermore, glutathione peroxidase 4 (GPX4) and ferroptosis suppressor protein 1 (FSP1) are antioxidant enzymes that prevent lipid oxidation and act in an inhibitory manner against ferroptosis [4]. The association between 4-HNE accumulation, as well as GPX4 and FSP1 expression, and cancer has been investigated in various cancers, including gastric cancer and hepatocellular carcinoma, and has attracted attention as a potential new therapeutic strategy [5,6,7,8]. For example, hepatocellular carcinoma has been reported to have a worse prognosis with lower accumulation of 4-HNE and higher expression of GPX4 [9]. On the other hand, in diffuse large B-cell lymphoma (DLBCL), lower expression of GPX4 and FSP1 is associated with a worse prognosis [10]. In lung cancer, squamous cell carcinoma has been reported to have a worse prognosis with lower accumulation of 4-HNE and lower expression of GPX4 and FSP1 [11]. In terms of GPX4 and FSP1 expression levels and ferroptosis induction, it is known that the lower the expression levels of these factors, the more likely ferroptosis is induced by depletion of the two factors [12]. However, the degree of accumulation and expression of lipid peroxidation markers and cancer prognosis in lung adenocarcinoma have not been investigated.

In this study, we evaluated the prognostic impact of lipid peroxidation markers and regulators involved in ferroptosis in lung adenocarcinoma. Additionally, we conducted cell experiments to examine the impact of these factors and regulators on cell death.

## 2. Materials and Methods

### 2.1. Patient Selection

The study comprised 207 patients who underwent resection surgery at Tokyo Medical and Dental University Hospital for stage I–IV lung adenocarcinoma between June 2014 and October 2019. The median follow-up period was 44.8 months (0.23–108.0 months). Clinicopathologic data were retrospectively obtained from patients’ medical records and pathology reports. Two pathologists produced pathologic diagnoses based on the lung tumor classification system used by the World Health Organization (WHO). Cases with suspected metastases from the primary cancer to other organs were excluded. For every patient, informed consent was acquired. The Tokyo Medical and Dental University Clinical Trial Review Committee granted ethical approval (No. M2000-1706).

### 2.2. Immunohistochemistry

Samples of tumors were embedded in paraffin and fixed in 10% neutral buffered formalin. Sections measuring 4 μm were then stained and deparaffinized. The sections underwent heat-induced antigen retrieval, 3% hydrogen peroxide endogenous peroxidase blocking, and normal serum blocking. After that, they were incubated with primary antibodies against GPX4, FSP1, and 4-HNE for a whole night at 4 °C. The ABC kit (Vector Laboratories, Burlingame, CA, USA) and diaminobenzidine (Nichirei Bioscience, Tokyo, Japan) were used to detect GPX4, FSP1, and 4-HNE. Nuclear staining was carried out using hematoxylin (Muto Pure Chemicals Co., Ltd., Tokyo, Japan). Detailed immunohistochemical information is provided in Table 1.

### 2.3. Evaluation of Staining Intensity and Stratification

**Figure 1:**
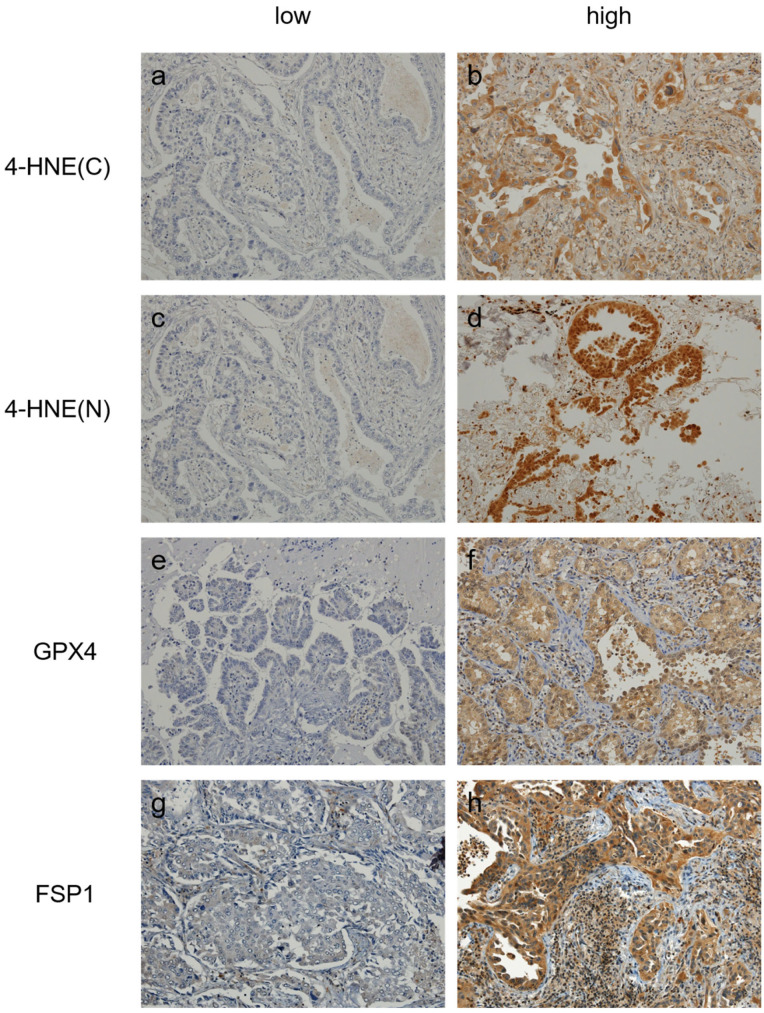
Images of lung adenocarcinoma patients’ immunostaining results for 4-HNE, GPX4, and FSP1. (**a**) Low cytoplasmic accumulation of 4-HNE. (**b**) High cytoplasmic accumulation of 4-HNE. (**c**) Low nuclear accumulation of 4-HNE. (**d**) High nuclear accumulation of 4-HNE (**e**) Low expression of GPX4. (**f**) High expression of GPX4. (**g**) Low expression of GPX4. (**h**) High expression of FSP1. 4-HNE(C): Accumulated 4-HNE in the cytoplasm. 4-HNE(N): Accumulated 4-HNE in the nucleus.

Immunostaining was performed to evaluate the accumulation of 4-HNE and the expression levels of GPX4 and FSP1 in lung adenocarcinomas. Evaluation of specimen staining was performed independently by two experienced pathologists. The accumulation of 4-HNE was evaluated in the cytoplasm and nucleus, and the results were categorized into four groups. A histochemical score (H-score) was calculated as the product of staining intensity and percentage of positive cells (range: 0–200). The median H-score was calculated for each group, which was then divided into two groups: those with scores above or equal to median and those below the median (Figure 1).

### 2.4. Clinicopathological Analysis

In addition to age, sex, smoking history, pathologic lung cancer stage (pStage), lymphovascular invasion, vascular invasion, and pleural invasion, several clinicopathologic parameters were evaluated, including the time from surgery to recurrence in patients treated with chemotherapy and treatment efficacy. The association of these parameters with 4-HNE accumulation and GPX4/FSP1 expression was analyzed. Furthermore, univariate and multivariate analyses were conducted based on the clinical parameters and staining results to evaluate their potential as independent prognostic factors.

### 2.5. Cell Lines and Culture Conditions

A549 and ABC-1 human lung adenocarcinoma cells were used for in vitro analyses. These cells were obtained from the Japanese Collection of Research Bioresources. They were cultured in D-MEM (high glucose) medium containing L-glutamine, phenol red (Fujifilm Wako Pure Chemicals, Osaka, Japan), 10% fetal bovine serum, and 1% penicillin-streptomycin. The cells were passaged every 3–4 days at a ratio of 1:10.

### 2.6. Western Blotting

Proteins were extracted from various lung adenocarcinoma cells and expression levels of GPX4 and FSP1 were confirmed. Whole cell lysates were separated on Any kD Mini-Protian TGX Stain-Free™ gels (BIO-RAD, Hercules, CA, USA, #4568125) and transferred to a PVDF membrane. Membranes were blocked with Bullet Blocking One for Western Blotting (NACALAI TESQUE, Inc., Kyoto, Japan, 13779-01). Primary antibodies were the following at a 1:1000 dilution: Anti-Glutathione Peroxidase 4 antibody (EPNCIR144) (Abcam, Cambridge, UK, ab125066), anti-AIFM2 antibody Rabbit host antibody (Merck KGaA, Darmstadt, Germany, HPA042309), β-Actin (13E5) Rabbit mAb (Cell Signaling Technology, Danvers, MA, USA, #4970). Secondary antibodies were Anti-Rabbit IgG, HRP-Linked Whole Ab Donkey (Cytiva, Tokyo, Japan, GE Healthcare NA934) at a 1:5000 dilution. Membranes were detected using Clarity Western ECL Substrate (BIO-RAD) and ChemiDoc™ MP (BIO-RAD).

### 2.7. Cell Proliferation Under 4-HNE Exposure

Cell proliferation of A549 and ABC-1 cells was evaluated under 4-HNE (Merck KGaA, Darmstadt, Germany) exposure. 4-HNE was diluted in 100% ethanol. Each cell was seeded in 96-well culture plates at a density of 1 × 10^3^ cells/well. After 24 h of incubation at 37 °C, the cells were exposed to 4-HNE at the following concentrations: 0, 20, and 40 µM. At 0, 24, 48, and 72 h after 4-HNE exposure, cell growth was confirmed using the Cell Counting Kit-8 (DOJINDO LABORATORIES, Kumamoto, Japan). Cells were incubated at 37 °C for 1.5 h after the addition of the Cell Counting Kit-8 reagent, and the absorbance was measured at 450 nm using an ELx808 microplate reader (Agilent Technologies, Santa Clara, CA, USA).

### 2.8. Cell Death Analysis Under 4-HNE and Cisplatin Exposure

Cell death of A549 and ABC-1 cells exposed to 4-HNE and cisplatin (Fujifilm Wako Pure Chemicals, Osaka, Japan), an anticancer drug widely used in chemotherapy for lung cancer, was evaluated. Each cell was seeded in 6-well culture plates at a density of 5 × 10^4^ cells/well. After 24 h of culture at 37 °C, cells were exposed to 4-HNE (0 and 10 µM) and cisplatin (0 and 20 µM). Cells were harvested 48 h after exposure, and the number of live and dead cells was counted on a Countess II FL automated cell counter (Thermo Fisher Scientific, Waltham, MA, USA). The percentage of dead cells was then calculated.

### 2.9. Analysis of Cytotoxicity Under GPX4 and FSP1 Inhibitor Exposure

Cells were treated with a GPX4 inhibitor (RSL3; MedChemExpress, Tokyo, Japan) combined with an FSP1 inhibitor (iFSP1; R&D System, Minneapolis, MN, USA), as well as a GPX4 inhibitor (ML210; Sigma-Aldrich, St. Louis, MO, USA) combined with an FSP1 inhibitor (iFSP1). The percentage of dead cells was then determined. Synergistic effects of treatment with both reagents were also investigated: A549 and ABC-1 cells were seeded in 96-well culture plates at a density of 3 × 10^3^ cells/well. After 24 h of incubation at 37 °C, the cells were treated with RSL3 at final concentrations of 0, 62.5, 125, and 250 nM combined with iFSP1 at concentrations of 0, 1.25, 2.5, and 5 µM. Additionally, ML210 was applied at concentrations of 0, 100, 200, and 400 µM combined with iFSP1 at 0, 1.25, 2.5, and 5 µM. The cytotoxicity LDH Assay Kit-WST (DOJINDO LABORATORIES, Kumamoto, Japan) was used to measure cell damage 24 h after exposure. The reaction was stopped by adding 50 µL of stop solution after incubation in the dark for 30 min at room temperature. Cell damage was measured by measuring the absorbance at 490 nm using an ELx808 microplate reader.

### 2.10. Detailed Analysis of Cell Death Using Cell Death Inhibitors

The effects of different cell death inhibitors on cell death induced by RSL3 and iFSP1 were investigated in A549 and ABC-1 cells. A combination of 250 nM RSL3 and 5 µM iFSP1 was simultaneously administered with the following cell death inhibitors for 24 h: 25 µM Z-VAD-FMK (apoptosis inhibitor; Peptide Institute, Osaka, Japan), 20 µM Z-LEHD-FMK (apoptosis inhibitor; Medical Biological Laboratory, Nagoya, Japan), 100 µM necrostatin-1 (necrosis inhibitor; AdipoGen Life Sciences, San Diego, CA, USA), 1 µM chloroquine (autophagy-related cell death inhibitor; Sigma-Aldrich, St. Louis, MO, USA), 2 µM ferrostatin-1 (ferroptosis inhibitor; Sigma-Aldrich), 250 µM liprostatin (ferroptosis inhibitor; Sigma-Aldrich), 20 µM chloroquine-1 (ferroptosis inhibitor; AdipoGen Sigma-Aldrich), 20 µM deferoxamine (iron chelator and ferroptosis inhibitor; Sigma-Aldrich), and 100 µM vitamin E (antioxidant and ferroptosis inhibitor; FujiFilm-WAKO, Osaka, Japan). The same Cytotoxicity LDH Assay Kit-WST was used for measurements as above.

### 2.11. Statistical Evaluation

Fisher’s exact test was performed and phi coefficients were calculated to evaluate the accumulation of 4-HNE in the cytoplasm and nucleus and the association between GPX4 and FSP1 expression levels. Overall survival (OS) was defined as the time from the date of surgery to the date of last follow-up or death. Recurrence-free survival (RFS) was defined as the time from the date of surgery to the date of last follow-up or the date when cancer recurrence was diagnosed by CT or PET scan. For OS and RFS, survival curves were plotted by Kaplan–Meier method, and differences of OS and RFS between the high and low expression groups of 4-HNE, GPX4, and FSP1 were evaluated using the log-rank test. Cox proportional hazards regression models were used to perform univariate and multivariate analyses to assess independent prognostic factors for OS and RFS. Student’s *t*-test was used for all in-vitro analyses, and Dunnett’s test was used for comparisons among the three groups in experiments examining the proliferative potential and cell death of 4-HNE. At *p* < 0.05, substantial changes were deemed statistically significant. IBM SPSS 22 (IBM Corp., Armonk, NY, USA) and GraphPad Prism version 10.0 (GraphPad Software Inc., San Diego, CA, USA) were used for statistical analyses.

## 3. Results

### 3.1. Measurement of H-Scores for 4-HNE, GPX4 and FSP1 in Immunostaining and Stratification of Cases

To stratify cases according to staining intensity, H-scores for 4-HNE (cytoplasmic and nuclear), GPX4, and FSP1 were evaluated. The median H-score was calculated for each antibody used, and patients were classified into the high expression group if their H-score was equal to or above the median. If the H-score was below the median, patients were classified into the low expression group. Histograms of H-scores for each staining result are shown in Supplementary Figure S1a–d. The median accumulation of 4-HNE in the cytoplasm was 100, with 110 and 97 patients classified in the high and low accumulation groups, respectively. Accumulation of 4-HNE was also observed in the nucleus, but the majority of patients did not show nuclear accumulation. For GPX4, the median H-score was 30, with 118 and 89 patients classified in the high and low expression groups, respectively. Similarly, for FSP1, the median H-score was 140, with 105 and 102 patients classified in the high and low expression groups, respectively. All subsequent studies were based on this classification.

### 3.2. Assocation of 4-HNE, GPX4, and FSP1 Expression Levels

To determine whether there was an association between the accumulation of 4-HNE and the expression levels of GPX4 and FSP1, we classified each factor into high and low groups and performed Fisher’s exact test and calculated phi coefficients (Table 2). 4-HNE was not associated with GPX4, and similarly GPX4 was not associated with FSP1. An association was found between the accumulation of 4-HNE in the cytoplasm and the expression level of FSP1, but the phi coefficient was 0.352, a weak correlation. An association was also found between cytoplasmic and nuclear levels of 4-HNE, but the phi coefficient was weak at 0.265.

### 3.3. Prognostic Analysis of Lipid Peroxidation Regulators and Markers in Lung Adenocarcinoma

**Figure 2:**
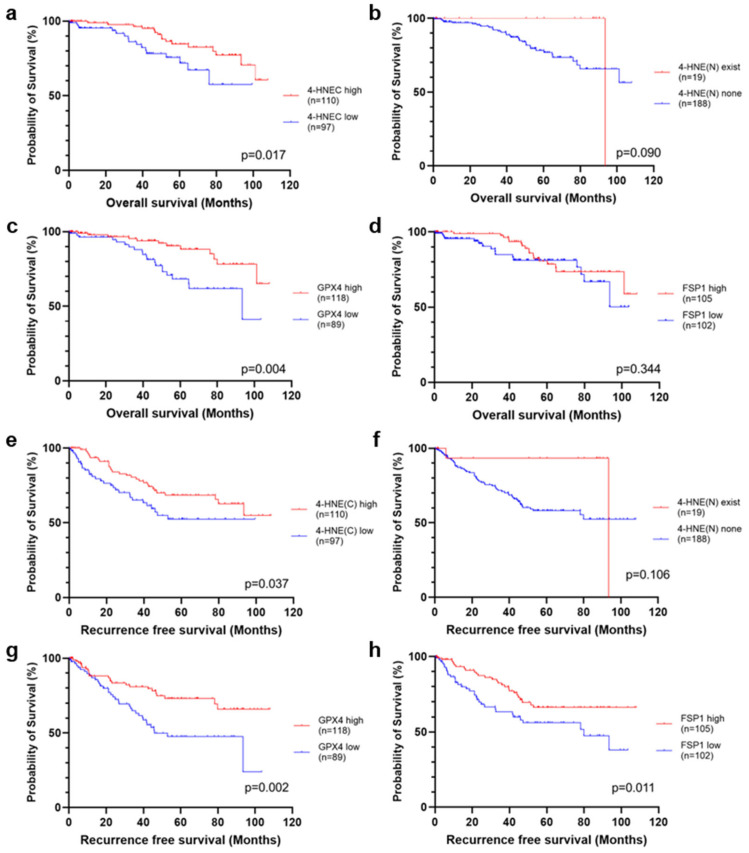
Kaplan–Meier survival curves showing overall survival and recurrence-free survival in light of the cytoplasmic and nuclear accumulation of 4-HNE and the expression levels of GPX4 and FSP1 in lung adenocarcinoma. (**a**) Overall survival depending on the accumulation of 4-HNE in the cytoplasm. (**b**) Overall survival depending on the accumulation of 4-HNE in the nucleus. (**c**) Overall survival depending on the expression of GPX4. (**d**) Overall survival depending on the expression of FSP1. (**e**) Recurrence free survival depending on the accumulation of 4-HNE in the cytoplasm. (**f**) Recurrence free survival depending on the accumulation of 4-HNE in the nucleus. (**g**) Recurrence free survival depending on the expression of GPX4. (**h**) Recurrence free survival depending on the expression of FSP1.

To evaluate the association between lipid peroxidation regulators and markers and prognosis in lung adenocarcinoma, prognostic analysis was performed for each factor. Kaplan–Meier survival curve analysis showed that high accumulation of 4-HNE in the cytoplasm and high expression of GPX4 were associated with a good prognosis (*p* = 0.017 and *p* = 0.004, respectively) (Figure 2a,c). On the other hand, accumulation of 4-HNE in the nucleus (*p* = 0.090) and expression of FSP1 (*p* = 0.344) were not clearly associated with prognosis (Figure 2b,d).

Similarly, univariate analysis was also performed for RFS. Again, low accumulation of 4-HNE in the cytoplasm and low expression of GPX4 were associated with worse prognosis (*p* = 0.037 and *p* = 0.002, respectively) (Figure 2e,g). In addition, low expression of FSP1 was also associated with unfavorable prognosis (*p* = 0.011) (Figure 2h), whereas nuclear accumulation of 4-HNE had no clear effect on RFS or OS (*p* = 0.106) (Figure 2f).

### 3.4. Association Between Accumulation/Expression Levels and Clinicopathological Factors

We evaluated whether an association existed between the accumulation and expression levels of each factor and clinicopathological factors (Table 3). The accumulation level of 4-HNE in the cytoplasm was associated with lymphatic invasion (*p* = 0.009) and vascular invasion (*p* = 0.016). Additionally, the accumulation level of 4-HNE in the nucleus was associated with vascular invasion (*p* = 0.011), and the expression level of FSP1 was associated with pleural invasion (*p* = 0.024). However, none of these were associated with the stage of disease.

### 3.5. Univariate and Multivariate Analysis

Cox univariate analysis was used to evaluate the association between OS and 10 parameters: age, sex, disease stage, lymphatic invasion, vascular invasion, pleural invasion, cytoplasmic and nuclear accumulation of 4-HNE, and expression levels of GPX4 and FPS1 (Table 4). As a result, in addition to disease stage, lymphatic invasion, vascular invasion, and pleural invasion, cytoplasmic 4-HNE accumulation, and the expression level of GPX4 were found to be associated with OS. Furthermore, multivariate analysis of each factor revealed that, in addition to disease stage, low expression of GPX4 was an independent poor prognostic factor (Table 5).

### 3.6. Inhibition of Cell Proliferation in Lung Adenocarcinoma Cell Lines A549 and ABC-1 by 4-HNE

In this study, fisher’s test showed no association between 4-HNE and GPX4 or between 4-HNE and FSP1, suggesting that 4-HNE itself is toxic to cells. Therefore, in vitro experiments were conducted to evaluate the molecular and cellular mechanisms of 4-HNE in the actual cellular environment of lung adenocarcinoma.

**Figure 3:**
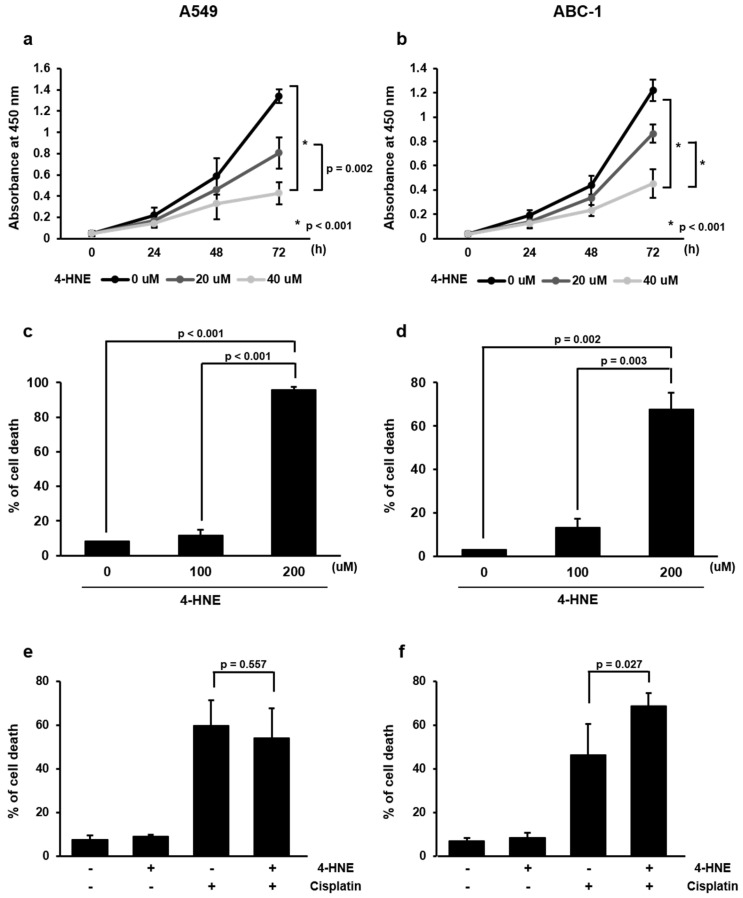
(**a**) Curve graph contrasting the dose-dependent impact of 4-HNE exposure on A549 cell proliferation. (**b**) Curve graph contrasting the dose-dependent impact of 4-HNE exposure on ABC-1 cell proliferation. (**c**) Bar graph showing the percentage of cell death by concentration when A549 cells were exposed to high concentrations (0 vs. 100 vs. 200 µM) of 4-HNE. (**d**) Bar graph showing the percentage of cell death by concentration when ABC-1 cells were exposed to high concentrations (0 vs. 100 vs. 200 µM) of 4-HNE. (**e**) Bar graph showing how cisplatin (20 µM) affects A549 cells exposed to 4-HNE (10 µM) versus those that are not exposed in terms of cell death. (**f**) Bar graph showing how cisplatin (20 µM) affects ABC-1 cells exposed to 4-HNE (10 µM) versus those that are not exposed in terms of cell death.

The lung adenocarcinoma cell lines A549 and ABC-1 were used to evaluate the degree of proliferation of these cells in the presence of 4-HNE. When A549 cells were exposed to 4-HNE at concentrations of 0, 20, and 40 µM, cell proliferation was inhibited at 20 µM and 40 µM. Moreover, higher concentrations of 4-HNE led to increased inhibition of cell proliferation (0 vs. 20 µM; *p* = 0.002, and 0 vs. 40 µM; *p* < 0.001) (Figure 3a). The same experiment was performed with ABC-1 cells, and the results showed that cell proliferation was similarly inhibited at 20 µM and even more so at 40 µM (0 vs. 20 µM; *p* = 0.001, and 0 vs. 40 µM; *p* < 0.001) (Figure 3b). Observations were made in 24-h increments up to 72 h, and inhibition was enhanced at 72 h.

### 3.7. Evaluation of the Effect of 4-HNE on Induction of Cell Death in Lung Adenocarcinoma Cells

To investigate the effect of 4-HNE on apoptosis, we performed the following experiments. First, we analyzed whether 4-HNE induces cell death at high concentrations. To further evaluate the cytotoxic potential of 4-HNE, lung adenocarcinoma cells were exposed to high concentrations of 4-HNE and the percentage of cell death was observed at 48 h. The percentage of cell death increased in a dose-dependent manner in A549 cells (0 vs. 200 µM; *p* < 0.001, 100 vs. 200 µM; *p* < 0.001) (Figure 3c), A similar trend was observed in ABC-1 cells (0 vs. 200 µM; *p* = 0.002, 100 vs. 200 µM; *p* = 0.003) (Figure 3d). To further investigate the effect of cisplatin on apoptosis induction at low concentrations of 4-HNE, the following experiment was designed. Cells were divided into 4-HNE-exposed and non-exposed groups, and each group was subsequently treated with cisplatin to check the degree of cell death in response to cisplatin. The results showed no significant difference in the cell death rate between the 4-HNE-exposed and non-exposed groups in A549 cells (*p* = 0.557) (Figure 3e). However, in ABC-1 cells, the cell death rate in the 4-HNE-exposed group was significantly higher than that in the non-exposed group (*p* = 0.027) (Figure 3f).

### 3.8. Synergistic Induction of Non-Apoptotic Cell Death in Lung Adenocarcinoma Cells by Combined Inhibition of GPX4 and FSP1

Resistance to chemotherapy is one of the factors contributing to poor prognosis in cancers, including lung adenocarcinoma [13]. Chemotherapy mainly aims to induce apoptosis in cancer cells, but inducing cell death other than apoptosis as a treatment for chemotherapy-resistant cancers may contribute to improving prognosis. Clinicopathological studies showed that low expression of GPX4 and FSP1 was associated with a relatively poor prognosis. Moreover, among them, GPX4 was a poor prognostic factor in both OS and RFS. Among lung adenocarcinoma cells, A549 and ABC-1 cells are cells with relatively lower GPX4 expression than other lung adenocarcinoma cells such as VMRC-LCD and RERF-LC-MS cells (Supplementary Figure S2), which may be relatively well suited to deplete GPX4 and FSP1 and induce ferroptosis. We used these cells to induce ferroptosis by depleting GPX4 and FSP1. Therefore, we examined whether ferroptosis could be induced by depleting GPX4 and FSP1 in lung adenocarcinoma cells.

**Figure 4:**
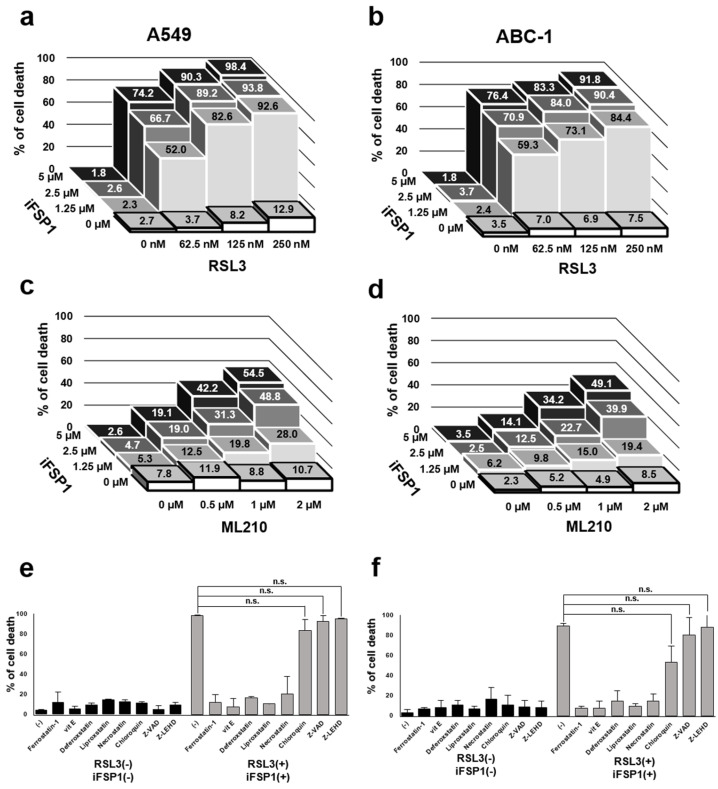
Induction and suppression of cell death by GPX4 and FSP1 inhibitors in A549 and ABC-1 cells at 48 h. (**a**) Percentage of cell death after RSL3 (0, 62.5, 125, and 250 nm) and iFSP (0, 1.25, 2.5, and 5 µm) treatment in A549 cells. (**b**) Percentage of cell death after RSL3 (0, 62.5, 125, and 250 nm) and iFSP (0, 1.25, 2.5, and 5 µm) treatment in ABC-1 cells. (**c**) Percentage of cell death after ML210 (0, 0.5, 1, and 2 µm) and iFSP (0, 1.25, 2.5, and 5 µm) treatment in A549 cells. (**d**) Percentage of cell death after ML210 (0, 0.5, 1, and 2 µm) and iFSP (0, 1.25, 2.5, and 5 µm) treatment in ABC-1 cells. (**e**) percentage of cell death in A549 cells treated with 250 nM RSL3, 5 µM iFSP1, and cell death inhibitors. (**f**) Percentage of cell death in ABC-1 cells treated with 250 nM RSL3 and 5µM iFSP1, and cell death inhibitors. The following were used as cell death inhibitors: 250 µM Z-VAD-FMK, 20 µM Z-LEHD-FMK, 2 µM ferrostatin-1, 100 µM vitamin E, 20 µM deferoxamine, 250 µM liproxatin, 1 µM chloroquine, and 100 µM necrostatin-1.

First, to assess the potential of GPX4 and FSP1 as therapeutic targets, cell death rates of lung adenocarcinoma cells treated with GPX4 inhibitors (RSL3 and ML210) and FSP1 inhibitor (iFSP1) alone or in combination were determined. Neither agent alone induced substantial cell death, but the combinations of RSL3 with iFSP1 and ML210 with iFSP1 induced synergistically strong cell death (Figure 4a,b). In a comparison between GPX4 inhibitors, RSL3 induced more cell death than ML210 (Figure 4c,d). The standard deviation of the cell death percentages is shown in Supplementary Figure S3. Furthermore, this cell death was completely prevented by the ferroptosis inhibitor but was not inhibited by the apoptosis inhibitor, indicating that the cell death caused by GPX4 and FSP1 inhibition is non-apoptotic (Figure 4e,f).

## 4. Discussion

In this study, we investigated clinicopathological and molecular biological aspects of lung adenocarcinoma to determine whether the expression of GPX4 and FSP1, regulators of lipid peroxidation, and the accumulation of 4-HNE, a lipid peroxidation marker, are associated with prognosis and chemotherapy resistance.

A clinicopathological study of lung adenocarcinoma specimens showed that low GPX4 expression was an independent poor prognostic factor, while high FSP1 expression was associated with significantly better prognosis in terms of RFS. GPX4 and FSP1 are factors that mitigate lipid peroxidation through different mechanisms. Higher expression levels of these factors correspond to increased quenching of oxidative stress and enhanced protection against cytotoxicity. Similarly, high expression of GPX4 and FSP1 in cancer cells is presumed to reduce oxidative stress caused by cell proliferation and exposure to anticancer drugs, thereby promoting cancer cell survival and leading to a poor prognosis. Indeed, previous reports have shown that GPX4 expression is an independent poor prognostic factor in gastric cancer, acute lymphoblastic leukemia, and malignant lymphoma [5,14,15]. In addition, high expression of FSP1 was also an independent poor prognostic factor in DLBCL [15]. However, in this study, the group with high GPX4 expression in lung adenocarcinoma specimens had a better prognosis in terms of both OS and RFS. Other studies of other cancer types have also reported similar results to those of the current study, such as a higher survival rate in patients without distant metastasis in breast cancer with high GPX4 expression [16]. FSP1 has also been shown to be associated with a better prognosis in patients with endometrial and urothelial cancer [17,18]. One of the reasons for the results in this study, which are contrary to the clinicopathological hypothesis based on the functions of GPX4 and FSP1, may be the effect of oxidative stress, including lipid peroxidation, on the genome of cancer cells. Oxidative stress in cells is well known to cause DNA damage. Cancer cells that survive under high levels of oxidative stress are believed to accumulate more DNA damage, which may lead to additional genetic abnormalities that activate new oncogenes and tumor suppressor genes, ultimately contributing to the acquisition of drug resistance to chemotherapy [19,20]. Furthermore, in recent years oxidative stress has been shown to promote cancer development and resistance to chemotherapy by altering epigenetic mechanisms, such as DNA methylation, histone modifications, and non-coding RNA, and by altering gene expression control elements [21]. Under the condition that the accumulation of oxidative stress due to lipid peroxidation is more prevalent in the low expression group of GPX4 and FSP1 in lung adenocarcinoma, such genetic and epigenetic changes associated with oxidative stress could potentially create an advantageous state for cancer cells.

In this study, patients with higher accumulation of 4-HNE in cancer cells had a relatively better prognosis for both OS and RFS. Furthermore, exposure to 4-HNE in vitro inhibited cancer cell proliferation in a dose-dependent manner. 4-HNE is a metabolic product of lipid peroxidation and has various physiological functions in cells [22]. Regarding its function in cell proliferation, 4-HNE affects the proliferation and differentiation of hematopoietic stem cells (HSCs), and exposure of HSCs to 4-HNE inhibits cell proliferation in a dose-dependent manner [23]. 4-HNE is also detected at relatively high steady-state levels in various human disease processes and has been reported to regulate cell proliferation through interference with cyclin and protein kinase activity and the apoptosis machinery [24]. Furthermore, exposure to 4-HNE has been reported to induce a decrease in intracellular glutathione and an increase in ROS, resulting in the induction of ERK1/2-mediated cell cycle arrest [25]. The accumulation of 4-HNE is believed to have a negative effect on proliferative cells, and the results of this study may be due to such a mechanism. On the other hand, regarding cell death, 4-HNE stimulates the intrinsic and extrinsic apoptotic pathways and induces apoptosis by interacting with factors such as p53, JNK, Fas, and mitochondrial regulators [2]. Furthermore, exposure to high concentrations of 4-HNE is known to induce cell death, including apoptosis [26]. If we consider that the accumulation of 4-HNE induces apoptosis in tumor cells, the accumulation of 4-HNE being associated with a tendency for patients to have a good prognosis is not surprising. However, the function of 4-HNE varies depending on the lipid composition pattern of the membrane of each tumor cell and the level of expression of detoxification enzymes and antioxidant proteins [2]. Moreover, tissue-specific aspects, such as differences in metabolic forms depending on the organ, further influence these dynamics [27]. Furthermore, the same DLBCL cell line has been reported to show different responses to FSP1 expression after exposure to 4-HNE, with some cell lines showing increased expression and others showing decreased expression. Furthermore, the same carcinoma shows different responses depending on the cell line [9]. In the current study, cell proliferation was inhibited by 4-HNE in both ABC-1 and A549 cells, but the response to 4-HNE and cisplatin administration differed. This may be influenced by differences in the metabolic capacity of 4-HNE due to differences in cell line characteristics, such as patterns of genetic abnormalities.

In vitro experiments showed that inhibition of either GPX4 or FSP1 alone caused little to no cell death in both A549 and ABC-1 cells, whereas inhibition of both led to rapid, presumably non-apoptotic cell death. Regarding the association between apoptosis induction in conventional chemotherapy and ferroptosis, high expression of MRP1 has been reported to decrease sensitivity to certain apoptosis while increasing sensitivity to ferroptosis [28]. This suggests that ferroptosis is a potentially effective therapeutic strategy for cancer cells that are resistant to or have acquired resistance to apoptosis. On the other hand, resistance to ferroptosis has been shown to be induced by BRCA1 deficiency, a gene associated with the risk of developing ovarian, breast, renal cell, and gastric cancers [29]. In these cancer cells, ferroptosis may be induced by regulating factors other than GPX4 and FSP1. Recent studies have reported that factors such as SLC7A11, NRF2, and xCT are involved in the process of ferroptosis in non-small cell lung cancer [30,31,32]. In addition, endogenous molecules, such as miRNA and lncRNA, have also been shown to control ferroptosis [33,34]. While GPX4 and FSP1 remain particularly important factors in the induction of ferroptosis, regulation of the abovementioned factors could lead to a therapeutic strategy using ferroptosis for a wider range of cancers.

The present study in lung adenocarcinoma showed similarities with previous studies in lung squamous cell carcinoma, particularly in terms of clinicopathological findings. That is, high accumulation of 4-HNE and a tendency for high expression of GPX4 and FSP1 indicate a relatively good prognostic pattern. Considering that the association with prognosis in DLBCL reported by Kawade et al. [10] and Kinowaki et al. [15] is different, the similarity of the two patterns is suggestive. In other words, it suggests that the organ and tissue environment specific to the lung may affect the mechanism of response to oxidative stress, including lipid peroxidation, in cancer. On the other hand, low expression of both GPX4 and FSP1 was associated with poor prognosis in lung squamous cell carcinoma [11], whereas low expression of GPX4 alone was associated with poor prognosis in lung adenocarcinoma. Gene mutations observed differ between lung adenocarcinoma and lung squamous cell carcinoma, with KRAS, EGFR, LPHN3, KEAP1, and TLR4 being relatively common in lung adenocarcinoma, whereas BAI3, FBXW7, GRM8, ERBB4, MUC16, and RUNX1T1 are common in lung squamous cell carcinoma [35]. Furthermore, patients with concurrent lung adenocarcinoma and lung squamous cell carcinoma have been reported to have similar gene mutations but significantly different gene expression and pathways [36]. By studying the molecular and tumor biological differences between lung adenocarcinoma and lung squamous cell carcinoma, which are different histological types of cancer in the same organ, it may be possible to understand the differences in the mechanisms involved in lipid peroxidation, responses to oxidative stress, and susceptibility to ferroptosis in different subtypes of cancer in other organs.

This study has several limitations. First, detailed histological subtypes of lung adenocarcinoma were not classified and analyzed. Second, gene mutations were not com-pared, and the association between gene mutations and the expression and accumulation of lipid peroxidation-related factors could not be clarified. Some genetic mutations are associated with resistance to chemotherapy, which may be related to recurrence rates and RFS. Third, in the experiment to verify ferroptosis by inhibiting GPX4 and FSP1, only GPX4 and FSP1 inhibitors were used in an in vitro experimental system, and verification by knockdown and knockout methods and in vivo effects are desired. Furthermore, the surgical procedures used were not standardized and the possibility of bias cannot be excluded. Fourth, there are insufficient detailed data on the chemotherapy administered, and the possibility that specific regimens or number of courses may have contributed to the relapse rate or duration of relapse has not been explored. The relationship between the efficacy of each chemotherapy regimen for patients who relapse and the expression of lipid peroxidation markers in lung cancer cells should also be evaluated. Fifth, the present study did not allow for a detailed examination of the expression levels of GPX4 and FSP1 per cell in lung adenocarcinoma tissue. This means that even if a patient is evaluated as having low expression of both GPX4 and FSP1, there may be cancer cells with relatively high expression of both GPX4 and FSP1, and these cells may affect the outcome of cancer treatment strategies with ferroptosis. In addition, since this was a retrospective study conducted at a single institution, prospective studies involving multiple institutions are warranted.

## 5. Conclusions

In this study, we demonstrated that factors related to ferroptosis can be biomarkers for the prognosis of lung adenocarcinoma and provided the basis for a new therapeutic strategy using ferroptosis induction by regulating GPX4 and FSP1 in lung adenocarcinoma cells. Based on these findings, we hope that further elucidation of the molecular mechanisms of ferroptosis in cancers, including lung cancer, will lead to the establishment of new cancer treatment strategies and save the lives of more cancer patients with poor prognosis.

## Figures and Tables

**Table 1:** Details of primary antibody conditions for GPX4, FSP1, and 4-HNE.

Antibody	Host	Type	Source	Antigen Retrieval	Buffer	Dilution	Method
4-HNE	Mouse	Monoclonal	Japan Institute for the Control of Aging	Microwave, 97 °C, 20 min	pH9.0	1:200	ABC
GPX4	Rabbit	Monoclonal	abcam	Microwave, 97 °C, 20 min	pH6.0	1:4000	ABC
FSP1	Rabbit	Polyclonal	ATLAS ANTIBODIES	Microwave, 97 °C, 20 min	pH9.0	1:200	ABC

**Table 2:** Association of 4-HNE with GPX4 and FSP1 Expression.

	**4-HNE(C) low**	**4-HNE(C) high**	***p* value**	**phi coefficient**
**GPX4 low**	39	50	0.483	−0.053
**GPX4 high**	58	60		
	**FSP1 low**	**FSP1 high**	***p* value**	**phi coefficient**
**GPX4 low**	44	45	0.889	0.011
**GPX4 high**	58	60		
	**4-HNE(C) low**	**4-HNE(C) high**	***p* value**	**phi coefficient**
**FSP1 low**	66	36	<0.001	0.362
**FSP1 high**	31	74		
	**4-HNE(N) none**	**4-HNE(N) exist**	***p* value**	**phi coefficient**
**GPX4 low**	79	10	0.467	−0.062
**GPX4 high**	109	9		
	**4-HNE(N) none**	**4-HNE(N) exist**	***p* value**	**phi coefficient**
**4-HNE(C) low**	96	1	<0.001	0.265
**4-HNE(C) high**	92	18		
	**4-HNE(N) none**	**4-HNE(N) exist**	***p* value**	**phi coefficient**
**FSP1 low**	92	10	0.812	−0.024
**FSP1 high**	96	9		

**Table 3:** Association between clinicopathological characteristics and each lipid peroxidation marker.

**Characteristics**	**4-HNE(C)** **High** **(*n* = 110)**	**4-HNE(C) Low** **(*n* = 97)**	***p* Value**	**Characteristics**	**4-HNE(N)** **Expression** **(*n* = 19)**	**4-HNE(N) None** **(*n* = 188)**	***p* Value**
Age				Age			
<70	38	44	0.120	<70	5	77	0.325
≥70	72	53		≥70	14	111	
Gender				Gender			
Male	61	64	0.154	Male	8	117	0.138
Female	49	33		Female	11	71	
Stage				Stage			
I	76	57	0.146	I	16	117	0.077
II, III, IV	34	40		II, III, IV	3	71	
Lymphovascular invasion				Lymphovascular invasion			
None	101	76	0.009	None	17	160	1.000
Exist	9	21		Exist	2	28	
Vessel invasion				Vessel invasion			
None	76	51	0.016	None	17	110	0.011
Exist	34	46		Exist	2	78	
Pleural invasion				Pleural invasion			
None	78	64	0.457	None	15	127	0.438
Exist	32	33		Exist	4	61	
**Characteristics**	**GPX4 High** **(*n* = 118)**	**GPX4 Low** **(*n* = 89)**	***p* Value**	**Characteristics**	**FSP1 High** **(*n* = 105)**	**FSP1 Low** **(*n* = 102)**	***p* Value**
Age				Age			
<70	51	31	0.252	<70	40	42	0.672
≥70	67	58		≥70	65	60	
Gender				Gender			
Male	71	54	1.000	Male	62	63	0.776
Female	47	35		Female	43	39	
Stage				Stage			
I	81	52	0.144	I	73	60	0.114
II, III, IV	37	37		II, III, IV	32	42	
Lymphovascular invasion				Lymphovascular invasion			
None	99	78	0.551	None	93	84	0.239
Exist	19	11		Exist	12	18	
Vessel invasion				Vessel invasion			
None	74	53	0.667	None	71	56	0.065
Exist	44	36		Exist	34	46	
Pleural invasion				Pleural invasion			
None	83	59	0.549	None	80	62	0.024
Exist	35	30		Exist	25	40	

**Table 4:** Univariate analysis of the impact of patient characteristics on overall survival.

Variable	Category	Number of Patients	HR	95%CI	*p* Value
Age	<70	82	0.960	0.480, 1.920	0.908
	≥70	125			
Gender	Male	125	0.590	0.298, 1.170	0.147
	Female	82			
stage	I	133	4.638	2.158, 9.966	<0.001
	II, III, IV	74			
ly	-	177	3.453	1.096, 10.88	0.001
	+	30			
v	-	127	2.181	1.037, 4.588	0.021
	+	80			
pl	-	142	2.962	1.366, 6.423	0.001
	+	65			
4-HNE(C)	high expression	110	2.205	1.064, 4.569	0.017
	low expression	97			
4-HNE(N)	expression	19	4.686	1.686, 13.02	0.090
	no expression	188			
GPX4	high expression	118	2.690	1.338, 5.406	0.004
	low expression	89			
FSP1	high expression	105	1.387	0.692, 2.780	0.344
	low expression	102			

**Table 5:** Cox proportional hazard analysis of clinicopathological factors related to overall survival.

**Variable**	**Category**	**Number of** **Patients**	**HR**	**95%CI**	***p* Value**	**Variable**	**Category**	**Number of** **Patients**	**HR**	**95%CI**	***p* Value**
Age	<70	82	0.911	0.407, 2.039	0.821	Age	<70	82	0.901	0.403, 2.017	0.800
	≥70	125					≥70	125			
Gender	Male	125	0.835	0.361, 1.932	0.674	Gender	Male	125	0.856	0.371, 1.979	0.717
	Female	82					Female	82			
stage	I	133	3.271	1.304, 8.204	0.012	stage	I	133	3.237	0.831,5.962	0.012
	II, III, IV	74					II, III, IV	74			
ly	-	177	1.528	0.556, 4.204	0.411	ly	-	177	1.652	1.099,6.656	0.330
	+	30					+	30			
v	-	127	0.59	0.223, 1.564	0.289	v	-	127	0.516	0.189, 1.412	0.198
	+	80					+	80			
pl	-	142	2.042	0.811, 5.139	0.130	pl	-	142	2.125	0.841, 5.369	0.111
	+	65					+	65			
4-HNE(C)	high expression	110	1.042	0.468, 2.317	0.920	4-HNE(N)	expression	19	3.575	0.408, 31.36	0.250
	low expression	97					no expression	188			
**Variable**	**Category**	**Number of** **Patients**	**HR**	**95%CI**	***p* Value**	**Variable**	**Category**	**Number of** **Patients**	**HR**	**95%CI**	***p* Value**
Age	<70	82	0.794	0.348, 1.809	0.583	Age	<70	82	0.908	0.405, 2.034	0.815
	≥70	125					≥70	125			
Gender	Male	125	0.851	0.362, 2.000	0.711	Gender	Male	125	0.825	0.357, 1.908	0.654
	Female	82					Female	82			
stage	I	133	3.005	1.170, 7.715	0.022	stage	I	133	3.338	1.328, 8.391	0.010
	II, III, IV	74					II, III, IV	74			
ly	-	177	1.765	0.627, 4.972	0.282	ly	-	177	1.561	0.573, 4.258	0.384
	+	30					+	30			
v	-	127	0.562	0.206, 1.531	0.260	v	-	127	0.589	0.222, 1.568	0.290
	+	80					+	80			
pl	-	142	2.095	0.811, 5.408	0.126	pl	-	142	2.153	0.840, 5.517	0.110
	+	65					+	65			
GPX4	high expression	118	02.749	1.210, 6.246	0.016	FSP1	high expression	105	0.739	0.331, 1.649	0.460
	low expression	89					low expression	102			

## Data Availability

The study does not have any datasets that can be made publicly available.

## Supplementary Materials

The following supporting information can be downloaded at: https://www.mdpi.com/article/10.3390/cancers16223888/s1, Figure S1. The distribution of H-scores for each lipid peroxidation marker. Figure S2. Western blotting verified the expression levels of GPX4 and FSP1 in lung adenocarcinoma cells. Figure S3. Standard deviation of cell death ratio by GPX4 and FSP1 inhibitors in A549 and ABC-1 cells at 48 h.
