# Supplementary material for: GPX4 and FSP1 Expression in Lung Adenocarcinoma: Prognostic Implications and Ferroptosis-Based Therapeutic Strategies
Source: Cancers (Basel). 2024 Nov 20;16(22):3888. doi: 10.3390/cancers16223888 (PMC11592420; doi:10.3390/cancers16223888)
Supplement: Supplementary file 1 [file cancers-16-03888-s001.zip › cancers-3236554-supplementary.pdf]

Supplementary Materials: GPX4 and FSP1 Expression in Lung Adenocarcinoma: Prognostic Implications and Ferroptosis-Based Therapeutic Strategies

Hirotomo Takahara, Takumi Kanazawa, Haruna Oshita, Yoshinobu Tomita, Yuri Hananoi, Sachiko Ishibashi, Masumi Ikeda, Asuka Furukawa, Mayumi Kinoshita, Kurara Yamamoto, Yuki Kato, Hironori Ishibashi, Kenichi Okubo, Morito Kurata, Masanobu Kitagawa, Kenichi Ohashi and Kouhei Yamamoto

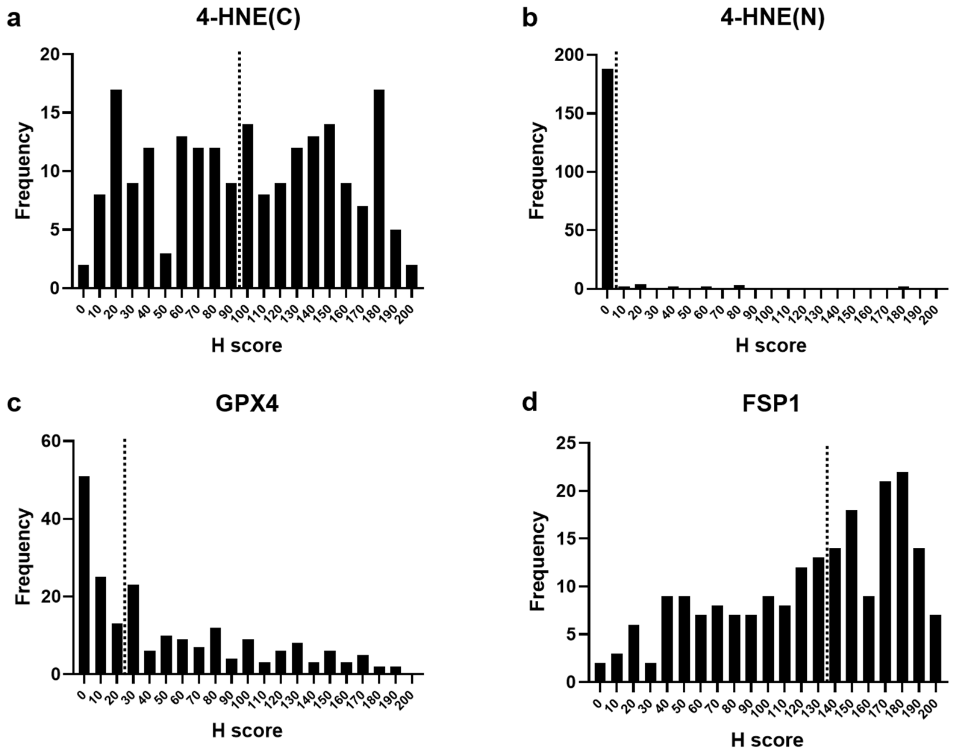

Figure S1. The distribution of H-scores for each lipid peroxidation marker. The dotted line in each graph indicates the borderline that classifies the high and low groups. (**a**) Histogram showing H-score of 4-HNE accumulation in cytoplasm. (**b**) Histogram showing H-score of 4-HNE accumulation in nucleus. (**c**) Histogram showing H-score of GPX4 expression. (**d**) Histogram showing H-score of FSP1 expression.

a **A549**

		RSL3			
		0 nM	62.5 nM	125 nM	250 nM
iFSP1	0 μM	1.763	0.013	3.635	1.812
	1.25 μM	1.425	3.102	1.805	0.146
	2.5 μM	0.715	0.228	0.298	4.172
	5 μM	2.458	3.368	6.186	2.089

b **ABC-1**

		RSL3			
		0 nM	62.5 nM	125 nM	250 nM
iFSP1	0 μM	2.071	0.333	0.015	0.399
	1.25 μM	0.087	17.297	15.232	11.956
	2.5 μM	1.616	13.444	10.802	6.884
	5 μM	1.108	8.962	12.572	9.561

c

		ML210			
		0 μM	0.5 μM	1 μM	2 μM
iFSP1	0 μM	3.547	6.653	4.359	1.191
	1.25 μM	0.239	0.623	5.515	8.170
	2.5 μM	1.507	2.375	0.473	1.958
	5 μM	2.931	2.245	5.690	3.825

d

		ML210			
		0 μM	0.5 μM	1 μM	2 μM
iFSP1	0 μM	2.291	1.546	1.863	1.804
	1.25 μM	0.749	0.128	9.307	12.898
	2.5 μM	0.880	1.341	5.006	2.823
	5 μM	2.945	0.190	7.370	2.678

Figure S2. Standard deviation of cell death ratio by GPX4 and FSP1 inhibitors in A549 and ABC-1 cells at 48 hours. (**a**) Standard deviation of the cell death ratio of A549 by RSL3 (GPX4 inhibitor) and iFSP1 (FSP1 inhibitor) (%). (**b**) Standard deviation of ABC-1 by RSL3 (GPX4 inhibitor) and iFSP1 (FSP1 inhibitor) (%). (**c**) Standard deviation of the cell death ratio of A549 by ML210 (GPX4 inhibitor) and iFSP1 (FSP1 inhibitor) (%). (**d**) Standard deviation of ABC-1 by ML210 (GPX4 inhibitor) and iFSP1 (FSP1 inhibitor) (%).

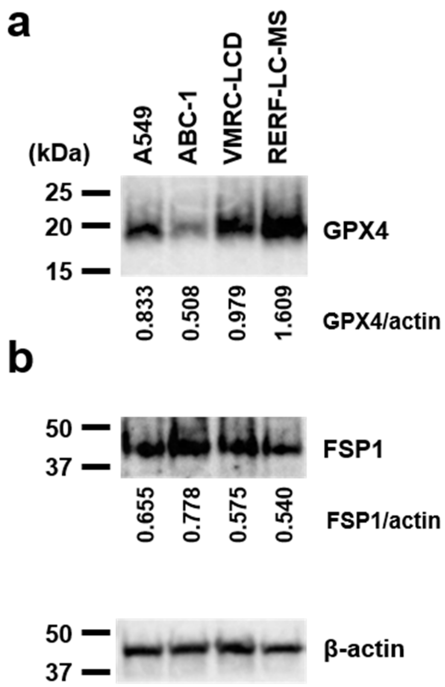

Figure S3. Western blotting verified the expression levels of GPX4 and FSP1 in lung adenocarcinoma cells. (**a**) GPX4 expression in A549 and ABC-1 cells was lower than in the other two cells. (**b**) FSP1 expression in A549 and ABC-1 cells was comparable to the other two cells.
